# The Relationship between Psychological Hardiness and Military Performance by Reservists: A Moderation Effect of Perceived Stress and Resilience

**DOI:** 10.3390/healthcare11091224

**Published:** 2023-04-25

**Authors:** Svajone Bekesiene, Rasa Smaliukienė, Rosita Kanapeckaitė

**Affiliations:** 1 General Jonas Zemaitis Military Academy of Lithuania, Silo 5a, LT-10322 Vilnius, Lithuania; 2 Institute of Psychology, Vilnius University, Universiteto g. 9/1, LT-01513 Vilnius, Lithuania

**Keywords:** perceived stress, personality, military performance, resilience, military reserve, tactical population, moderated mediation

## Abstract

The purpose of this study is to evaluate the effect of hardiness on the perceived military performance of reservists, i.e., young people who have full-time jobs in a civilian sector and perform military training as a part of their civic duty. We proposed the conceptual model with conditional indirect effects of the hardiness on personal military performance, where mediated moderation effects are observed from personality traits and variables important for military service: team cohesion, perceived stress, and psychological resilience. The final dataset was comprised of 384 self-reported paper–pencil questionnaires filled out by reserve soldiers, and PROCESS Macro 3.5 Model 7 and Model 14 were used for the analysis. The results revealed that perceived stress (Model 1) and psychological resilience (Model 2) have a statistically significant moderate mediating effect on the interlink between hardiness and performance when personality traits and team cohesion are taken into consideration. The change in R^2^ is statistically significant and explains how perceived stress and psychological resilience affect individuals. When psychological hardiness is low, the level of perceived stress has a statistically significant moderating effect, i.e., it reduces the effect of hardiness on performance. When comparing the effects of perceived stress and psychological resilience, the latter has a stronger moderating effect on performance. Specifically, the moderating effect of resilience was more evident in Model 2 (66.9% variance, r = 0.818) for the military performance of the reservists than the perceived stress in Model 1 (52.5% variance, r = 0.724). This means that resilience increases the accountability of Model 2 compared to Model 1 by 14.4%. We conclude that resilience training could statistically significantly increase the military performance of reserve soldiers as a tactical population.

## 1. Introduction

Most European countries are strengthening their military personnel by enlisting reservists on a voluntary and mandatory basis to increase the capability of their tactical population. Military reservists are young people who have full-time jobs in a civilian sector and perform military duties and military training on demand as part of their civil obligations. The transition from civilian life to military life and back again is iterative [1], so reservists have to use their hardiness after experiencing stress that comes from different environments. 

Hardiness is perceived as a psychological skill that gives positive effects after experiencing stress [2]. Hardiness among military personnel is found to be an exceptional personal quality that leads to adaptability and performance in an ambiguous environment during military operations [3]. In addition to having a challenge to balance their work and life, reservists try to overcome their work–life–duty conflict, where each area is time-consuming and challenging with regard to their tasks and activities [4,5,6]. In a military context, stressors can come in a variety of forms, including physical (e.g., extreme heat or cold, lack of sleep, and physical exhaustion) and psychological (e.g., fear, time pressure, heavy workload, uncertainty, and information overload) [7,8]. Therefore, it is important to understand what factors would create a supportive environment for these young people and help them balance their life and duty tasks.

Recently, the reservist service has attracted the attention of researchers as a unique environment where, on the one hand, there are many stressors [9,10,11], and on the other, stress coping skills are actively developed [10,12,13]. Stress resilience training helps soldiers deal with emotional and physiological reactions to stress and maintain optimal performance, even in high-pressure situations [14,15,16,17]. Psychological resilience developed during military training is found to be a protective factor for reservists that helps them maintain performance despite environmental stressors [18]. However, there are other factors that help soldiers remain engaged and continue to perform well in stressful conditions. Research indicates that personality traits contribute to military performance. Still, the findings on the relationship between personality and military performance remain controversial, with some studies showing no evidence of an interdependence [19], while others suggest personality as a predictor [20]. The effects are likely attributed to individual personality traits and their unique combinations, resulting in varying outcomes [21].

Furthermore, it should be mentioned that the military environment is unique in terms of team compositions and team cohesion as a form of psychological support in a stressful environment. Group cohesion in military training has been shown to increase resilience to stress [22,23] and reduce the level of stress among soldiers during military training [24,25]. Thus, given the specificity of the military environment, the effects of perceived stress, psychological resilience, personality traits, and team cohesion need to be considered to understand how resilience affects perceived military performance.

Despite extensive research carried out on hardiness in the military, the complexity of the hardiness–performance interrelationship among military reservists has not yet been elucidated. There is a lack of research that assesses this interrelationship, considering the effects of other individual and group characteristics, not only the direct effects of hardiness on military performance. This is an important research gap, taking into consideration that reservists are highly diverse in terms of their life experience that comes from their everyday civil life. Accordingly, with this study, our aim was to extend these findings by including other individual and group characteristics to better understand the complexity of the hardiness–performance interrelationship. We studied this interrelationship in a sample of Lithuanian Active Army Personnel Reserve (AAPR) soldiers after they completed a five-week reserve training course. In this paper, we test two models of the hardiness–performance interrelationship. Model 1 provides evidence on how perceived stress as a moderator affects the relationship between individual and group variables in military performance. As individual variables, personality traits were used in this research. The group variables were measured using group cohesion. Both group cohesion and personality traits, as indicators of small-group or individual behavior strategies, are expected to have a mediating effect on the hardiness–performance interrelationship. Model 2 explores the moderating effect of psychological resilience on the relationships mentioned above.

The purpose of this research is to evaluate the effect of hardiness on the perceived military performance of reservists, i.e., young people who have full-time jobs in a civilian sector and perform military training as a part of their civic duty. Our results have significant practical implications. They suggest that a certain level of hardiness, which is a stable personality characteristic over time, can effectively buffer the negative impact of perceived stress on military performance. Additionally, our findings provide evidence regarding the level of psychological resilience that should be developed during military exercises to enhance positive military performance. 

The rest of the paper is organized as follows: Section 2 provides a literature review and the hypothesis to be tested; Section 3 provides an overview of the research sample and research instrument used; Section 4 details the modeling results; and in Section 5, we discuss the findings of this study in the context of existing research, providing insights for future research directions.

## 2. Literature Review and Hypotheses

The military environment is perceived to be more stressful than the civilian one [7,8]; therefore, psychological hardiness is found to have a significant effect on the personal performance of a soldier [16]. Hardiness represents the inherent ability of people to manage stressful situations and turn stressful situations from potential disasters into opportunities for growth [26]. Hardiness is considered to be a stable personality characteristic over time [27] and is positively related to other personality traits that are anticipated to serve as protective factors against stress [28]. Considering the adverse living and operational conditions that soldiers have to endure during military training, such as stress, fatigue, anxiety, and fears [28,29], hardiness has been identified as one of the individual characteristics that can minimize the impact of these stressors among soldiers [30]. Taken together, the personal performance of a soldier may be determined by the hardiness of the solder; however, perceived stress and resilience, as well as personality traits and team cohesion in a military unit, should be included as important variables in a research model. 

### 2.1. Psychological Hardiness

Recent work on military performance indicates that psychological hardiness has a positive effect on the performance of recruits. Following Bartone & Bowles’ [18] research results, recruits with high levels of hardiness tend to rely on proactive stress coping strategies. Proactive problem-focused coping is found to not only decrease the level of stress, but to also have a positive effect on personal well-being [18], which is an overall highly desirable outcome. This could be explained by another study on military trainees, where hardiness was found to predict behavioral persistence, i.e., military trainees with higher hardiness were actively involved in military training for a longer period of time [31]. 

### 2.2. Psychological Resilience

The other psychological construct and determinant of military performance is psychological resilience. Resilience is described as the individual capacity to adapt and cope with adverse or unpleasant experiences [32]. Resilience can help reduce the impact of emotional or psychological disturbances. Studies in the military show that soldiers with high resilience are less likely to develop PTSD [33]. In general, resilience is also understood as a positive relationship between personality and the environment, increasing optimism and self-esteem, as well as stress-related growth [34]. In the context of military training, the key point is that resilience could develop as an individual capacity.

### 2.3. Personality Traits

Previous works focused on military training paid some attention to the personality traits of the soldiers. The researchers concluded that different types of personalities lead to different levels of perceived level of stress during military training [35]. It seems that personality can lead to different types of stress coping strategies, for example, greater levels of extraversion and conscientiousness were found to predict higher levels of problem solving and cognitive restructuring, whereas neuroticism was negatively associated with these factors [36]. Earlier research has identified a direct relationship between specific personality dimensions and resilience [37]. Despite a large body of research on personality and behavior in the face of life stressors, little is known about the role of personality when it comes to reservists and their behavior in a stressful military training environment [38]. The underlying mechanisms of reserve soldiers’ behavior have not been examined using all the Big Five dimensions that describe personality traits. Furthermore, it is unclear how these relationships may differ between different personality traits and the psychological resilience of reserve soldiers when soldiers operate in a stressful training environment.

### 2.4. Team Cohesion

In addition, numerous studies have confirmed the positive effects of team cohesion on individual performance in stressful situations in general [39,40] and in military environment in particular [17]. Furthermore, team cohesion was found to have a valuable impact on military training results and be beneficial for soldiers in managing perceived stress [41] and increasing their psychological resilience during military training [23]. Scholars argue that social support is vital to the physical and psychological health of soldiers in general and during military training in particular [42]. Team cohesion is a phenomenon that brings individuals together to achieve a goal, especially when they are under stress [43].

### 2.5. Perceived Military Performance

From an operational perspective, military effectiveness is understood as the interaction of the individual soldier with the system of assigned material resources [44]. On the level of a private soldier, military effectiveness is related to the soldier’s technical skills on the level that they are proficient in the military perceived specialty they are in [45]. Therefore, military performance is often measured as the individual skills obtained during training [46]. Thus, the personal performance of a reservist during military training can be described as the action of performing a task (mission) by using assigned military technologies and military equipment. Perceived performance is just as important an objective, as it is the basis of military morale. Without going into the study of military morale, we can only mention that morale is the conditioned quality of an individual soldier that compels the soldier to perform his duty, regardless of any hostile force or influence [47].

The perceived military performance of reservists is a strong motivator to continue military service and stay active and engaged during military training [9]. Perceived performance is a subjective self-measure of the success of performing military tasks, which leads to a positive attitude towards oneself as a soldier. Studies in sports provide evidence that perceived performance is related not only to motivation, but also to actual performance, for example, points scored [48]. Thus, positive self-evaluated performance is a precondition for achieving goals. 

**Figure 1:**
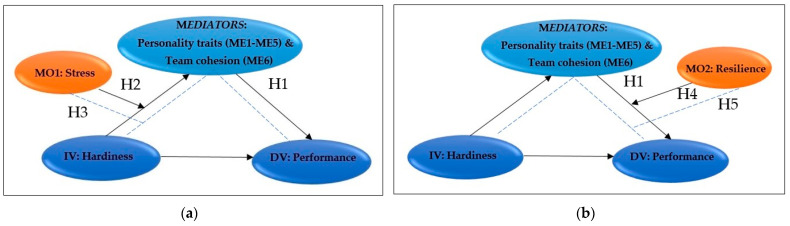
Hypothesized moderated mediation models of study: (**a**) Model 1 with perceived stress (MO1) as moderator, Independent Variable (IV): hardiness, and Dependent Variable (DV): performance; (**b**) Model 2 with psychological resilience (MO2) as moderator, Independent Variable (IV): hardiness, and Dependent Variable (DV): performance.

Taking these theoretical insights together, we constructed two conceptual models of influence that specify both individual and combined influences (see Figure 1). Using mediated moderation, we explain the conditional indirect effects of psychological hardiness on the perceived military performance of the reservists during military training. 

### 2.6. Mediating Effect of Personality Traits and Team Cohesion 

Following the methodological recommendations, the mediators explain the possible relationship between two variables, and are possible explanations for a relationship between the independent variable (IV: hardiness) and the dependent variable (DV: performance) [49]. In our case, since personality traits are considered to remain mainly stable throughout life [50] and team cohesion refers to perceived organizational support and associates’ support, we used mediation analyses to explain non-directional relationships between hardiness and military performance. Following this, personality traits and team cohesion work as mediators; consequently, we formulated the hypothesis of relationships among hardiness, personality traits, team cohesion, perceived stress or psychological resilience, and perceived military performance:

**Hypothesis** **H1.** *Reserve solders’ five personality traits and their team cohesion will mediate the relationships between hardiness and perceived military performance.*

We tested this hypothesis by using a sub-hypothesis for each indicator: the mediating effect of conscientiousness was tested using Hypothesis H1a, emotional stability using Hypothesis H1b, extraversion using Hypothesis H1c, agreeableness using Hypothesis H1d, openness to experiences using Hypothesis H1e, and team cohesion using Hypothesis H1f. The detailed information is provided in Table A1 (Appendix A). 

### 2.7. Moderating Effect of Perceived Stress (Model 1) and Psychological Resilience (Model 2)

Following the methodological recommendations, the moderators affect the magnitude of the effect of the independent variable (hardiness) on the dependent variable (performance) [51]. Following the literature, perceived stress has a significant negative effect on performance [52], while psychological resilience, in contrast, has a statistically significant positive effect on performance [51]. Both of these variables are found to be strong moderators in various psychological models [48,49,52]. Accordingly, we formulate the hypothesis of moderating effects:

**Hypothesis** **H2.** *Perceived stress will play a moderating role in the relationship between hardiness and reserve soldiers’ personality traits and their team cohesion.*

We tested this hypothesis by using sub-hypotheses for each indicator: the moderating effect on conscientiousness was tested using Hypothesis H2a, emotional stability using Hypothesis H2b, extraversion using Hypothesis H2c, agreeableness using Hypothesis H2d, openness to experiences using Hypothesis H2e, and team cohesion using Hypothesis H2f. The detailed information is provided in Table A1 (Appendix A).

**Hypothesis** **H3.** *Perceived stress will have a moderating effect on the mediating role of reserve soldiers’ personality traits and team cohesion.*

Similarly to the previous hypothesis, we tested this hypothesis using sub-hypotheses H3a–H3f. The detailed information is provided in Table A1 (Appendix A). We expect that the mediated relationship will be weaker under high perceived stress than under low perceived stress.

**Hypothesis** **H4.** *Psychological resilience will play a moderating role in the relationship between perceived performance and reserve soldiers’ personality traits and team cohesion.*

Accordingly, we developed sub-hypotheses H4a–H4f. The detailed information is provided in Table A1 (Appendix A).

**Hypothesis** **H5.** *Psychological resilience has a moderating effect on the mediating role of reserve soldiers’ personality traits and team cohesion.*

Accordingly, we developed sub-hypotheses H5a–H5f. The detailed information is provided in Table A1 (Appendix A). We expect that the mediated relationship will be stronger under high resilience than under low.

Following these hypotheses, we constructed two research models (see Figure 1). Hypothesized Model 1 (Figure 1a or Figure A2a in Appendix B) is constructed to test how stress moderates the effect of psychological hardiness on perceived military performance and what mediating effects contribute to personality traits and team cohesion. Hypothesized Model (Figure 1b or Figure A2b in Appendix B) is constructed to test what moderating effect has resilience on these interdependencies.

## 3. Materials and Methods

### 3.1. Study Participants

We used simple random sampling and delivered 400 questionnaires to randomly selected Active Army Personnel Reserve (AAPR) soldiers who had been called up for a 5-week reserve training course, which began in January and ended in February 2022. There were 390 completed paper–pencil questionnaires (97.5% response rate). The check for quality and missing value analysis showed that there were 4% missing data in the collected questionnaires, so these questionnaires were removed from the data sample. The 384 questionnaires were the final dataset that was used for this investigation. The survey was carried out with men (100%). The demographic background of the reservists was provided by self-reported information. The age of the reserve soldiers ranged from 20 to 47 years, the average was 29.5 years (±SD = 4918), and most (81.7%) represented men with an age between 24 and 32 years. The education level of the soldiers in our study ranged from secondary education to a master’s degree. The surveyed soldiers were mainly single (69%). Additional demographic information is presented in Appendix A, Table A2.

The study was approved by the General Jonas Zemaitis Military Academy, Protocol No. PR-1815. Each participant was provided with information about the study; their voluntary participation and anonymity were ensured. 

### 3.2. Description of the Research Instrument

General and military-specific research instruments were used to measure the research variables. The instruments included measurements for five independent variables—psychological hardiness, perceived stress, personality traits, team cohesion, and psychological resilience—and one dependent variable, perceived military performance of the reservists. The instrument was composed of five scales.

Military Hardiness Scale. Psychological hardiness was measured as the degree to which reservists are committed, feel challenged, and have a sense of control over their work experiences in the military environment. Hardiness was measured by the 11-item Military Hardiness Scale [53]. The items reflected the three main components of psychological hardiness: military-specific commitment (e.g., I am proud to be in the army), reflecting a strong identity with the military; military-specific control (e.g., I have personal control over my performance), reflecting control and personal influence on results; military-specific challenge (e.g., I strive as hard as I can to be successful), reflecting how personal resources match professional challenges. Cronbach’s alpha for the selected 11 items of the Military Hardiness Scale in the present study was 0.904 (see Table 1).

Perceived Stress Scale. The level of perceived stress of the reserve soldiers was measured using a standardized Perceived Stress Scale [54,55]. Originally developed in 1983, PSS-10 is a classic stress assessment instrument of 10 items that helps to understand how events and changes affect perceived stress. The scale has a 5-point measurement, and the sum of 10 items (e.g., ‘You felt nervous and stressed?’, ‘You felt difficulties were piling up so high that you could not overcome them?’) can vary from 0 to 40, where the higher scores showing higher individual stress. The Cronbach alpha for these 10 items was 0.764 in the current study, similar to previously reported [56]. 

Big Five scale. Personality traits were measured using the Big Five scale of ten items, known as the five-factor model [57]. This personality assessment model helps identify five types of people’s behaviors [36]: the sociable and active person is an extravert; the softhearted and trusting person is associated with friendliness (in this scale, agreeableness); the organized and reliable person is conscientiousness; the calm and relaxed are emotionally stable; the curious and creative person is indicated as openness [57,58]. The original Ten Item Personality Measure (TIPI) [57] was first translated into Lithuanian, and a forward–backward translation was used to avoid confusion among the survey participants. Reliability is not applicable for this test [57], but mean and standard deviation scores were measured.

Team Learning Behavior Scale. The cohesion of the team of reserve soldiers was measured using the Team Learning Behavior Scale of 8 items [24], developed on the basis of the Group Cohesion Scale-Revised [59]. The Cronbach alpha for these 8 items (e.g., In my squad, we easily accomplish tasks) was 0.774 in the current study, similar to previously reported [24]. 

Perceived Military Performance Scale. We employed a perceived military performance scale to assess the soldiers’ performance across various areas during their military training, such as tactical field exercises, defensive operations, and defense in settlements, among others. Perceived military performance (DV) was measured using a list of 14 items that measured self-reported performance (e.g., ‘During the exercises I was capable: <…> of transmitting silent control signals’, etc.). Military performance was measured as perceived competence used in practice. The self-reported military performance was measured after completing military training. Cronbach’s alpha for these 14 items was 0.944 in the current study (see Table 1). 

### 3.3. Data Analysis

**Figure A1:**
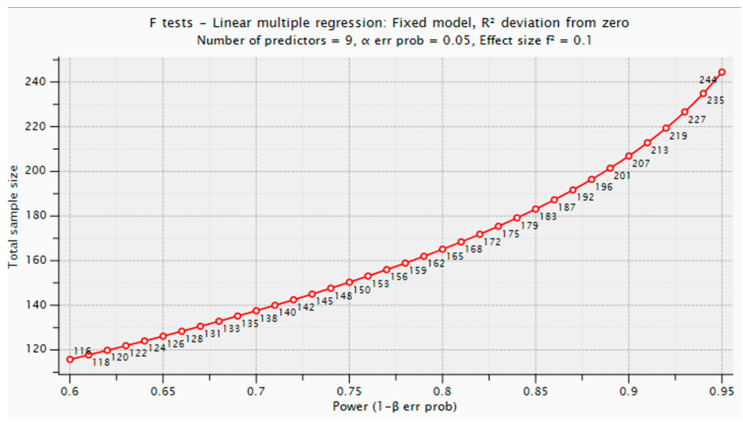
G∗Power v3.1.9.4 test result for data sample size estimation that is required to reach study statistical power.

The data were analyzed by SPSS 28v. The first G∗Power v3.1.9.4 test was conducted for the sample size evaluation of collected data. The statistical hypothesis testing was based on the 9 predictors that also accounted for the moderator, and with a significance level of 0.05, power of 0.95, and effect size of 0.1, it was indicated that a minimum sample size of 245 is required to reach statistical power (see Appendix B, Figure A1). Then, descriptive data analysis was conducted (i.e., M, ±SD). The internal consistency of the study variables’ scales was evaluated by Cronbach’s values. The statistical analysis conducted on construct convergent validity was achieved [60]. Pearson’s product moment correlation analysis was used to examine the relationships between the constructed ten study variables. In addition, we used Harman’s single-factor test and examined the possible variance of the common method [60]. 

**Figure A2:**
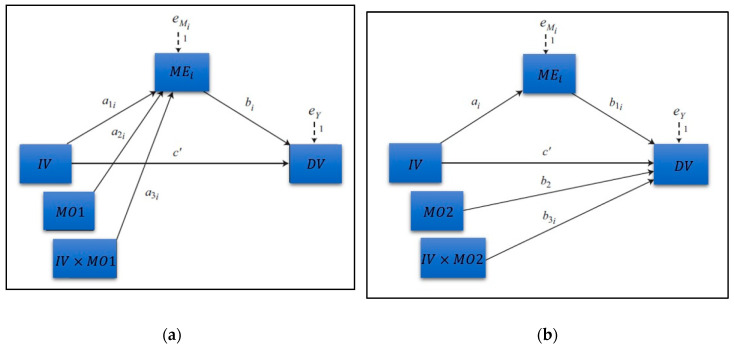
Statistical analysis diagrams for conditional indirect effect of independent variable (IV: military hardiness) on dependent variable (DV: perceived military performance): (**a**) Model 1, through mediators ${ME}_{i}=\left(a_{1i}+a_{3i}MO1\right)b_{i}$ and direct effect of $IV\to DV=c^{\prime }$; (**b**) Model 2, through mediators ${ME}_{i}=a_{i}\left(b_{1i}+b_{3i}MO2\right)$ and direct effect of $IV\to DV=c^{\prime }$.

The proposed theoretical models (Figure 1) were tested via moderated mediation analysis, also known as conditional indirect process modeling, using the PROCESS macro v3.5 developed by Hayes [61] for SPSS and the settings of Model 7 and Model 14. The designed models let us examine whether or not: (1) the effect of perceived stress on personality traits and team cohesion depends on perceived military performance; (2) the effect of resilience on personality traits and team cohesion depends on perceived military performance; (3) the effect of hardiness on perceived military performance depends on personality traits and team cohesion (see Appendix B, Figure A2). The selected study methodology supported the examination of direct and indirect effects of an independent variable (IV: military hardiness) on a dependent variable (DV: perceived military performance) through mediators (ME1, ME2, ME3, ME4, ME5, and ME6), as well as conditional effects that moderate these relationships for the first model by stress (MO1: perceived stress during the training period) and for the second model through a mediator (MO2: psychological resilience). Therefore, for Model 1, all hypotheses were tested concurrently by PROCESS macro v3.5 Model 7, and for Model 2, by PROCESS macro v3.5 Model 14. In addition, we followed the statistical test recommendations that are usually required for the examination of moderated mediation models [61]. Bias-corrected bootstrap confidence intervals (95% CI) were generated for conditional indirect effects at the low, average, and high level based on 5.000 bootstrap samples. Furthermore, all study variables were mean-centered.

## 4. Modeling Results

### 4.1. Study Constructs Evaluation

Before the causal relations assessment procedure, we first examined the relationship between our study constructs. Thus, the correlational analysis of perceived military performance (DV), hardiness (IV), conscientiousness (ME1), emotional stability (ME2), extraversion (ME3), agreeableness (ME4), openness to experiences (ME5), team cohesion (ME6), stress (MO1), and resilience (MO2) was conducted. 

The correlation investigation revealed that perceived military performance was positively correlated with hardiness (DV&IV, r = 0.554, *p* < 0.01) and psychological resilience (DV&MO2, r = 0.511, *p* < 0.001), while negatively correlated with perceived stress (DV&MO1, r = −0.259, *p* < 0.001). Hardiness was negatively correlated with perceived stress (IV&MO1, r = −0.476, *p* < 0.001) and with agreeableness, one of the personality traits (IV&ME4, r = −0.110, *p* < 0.05). On the other hand, hardiness was positively correlated with psychological resilience (IV&MO2, r = 0.292, *p* < 0.001). Additionally, a few personality traits were positively correlated with perceived military performance: conscientiousness (DV&ME1, r = 0.577, *p* < 0.01), emotional stability (DV&ME2, r = 0.350, *p* < 0.01), and openness to experiences (DV&ME5, r = 0.577, *p* < 0.01). The analysis results are presented in Table 2.

In addition, to prove that Common Methods Variance (CMV) cannot be a significant problem for this study, we used Harman’s one-factor test. The exploratory factor analysis conducted showed an appropriate value (0.867 > 0.8) for the Kaiser–Meyer–Olkin test. Furthermore, Bartlett’s sphericity test indicated with *p* < 0.001 that the data matrix (of correlations) is significantly different from an identity matrix. The explanatory power of the first factor was only 21.6% and was far from the recommended 50% threshold value [62].

### 4.2. Evaluation of Moderated Mediation on the Perceived Military Performance in Model 1

This study tested a moderating mediating effect of perceived stress (MO1) through personally based traits (ME1, ME2, ME3, ME4, ME5, and team cohesion (ME6)) on the perceived military performance (see Appendix B, Figure A2a, Model 1). How stress affects hardiness, personality traits, and team cohesion is presented in seven steps (see Table 3, Table 4 and Table 5). 

The conducted analysis shows that hardiness positively influenced soldiers’ personality traits, such as conscientiousness (IV**→**ME1, β = 0.726, *p* < 0.001) and emotional stability (IV**→**ME2, β = 0.492, *p* < 0.001), but negatively affected soldiers with agreeableness personality traits (IV**→**ME4, β = −0.279, *p* < 0.001) (see Table 3 and Table 4). 

Moreover, hardiness significantly and directly influenced the perceived military performance in this model (IV**→**DV, β = −0.273, *p* < 0.001). Consciousness also positively influenced perceived military performance (ME1**→**DV, β = 0.172, *p* < 0.001) (see Table 5, Model 1). Additionally, team cohesion showed a highly significant effect on perceived military performance (ME6**→**DV, β = 0.273, *p* < 0.001) (see Table 5, Model 1). Thus, conscientiousness (ME1) and team cohesion (ME6) can mediate the relationship between hardiness (IV) and perceived military performance (DV).

Thus, following Model 1, the hypothesis H2, if the perceived stress (MO1) will play a moderating role in the relationship between reserve soldiers’ personality traits and team cohesion, was examined. The significant conditional indirect effects were verified between hardiness and perceived military performance for reserve soldiers with personality traits such as conscientiousness (ME1×IV, F(1, 375) = 20.894, *p* < 0.001), emotional stability (ME2×IV, F(1, 375) = 9.425, *p* < 0.001), extraversion (ME3×IV, F(1, 375) = 30.863, *p* < 0.001), and team cohesion (ME6×IV, F(1, 375) = 46.850, *p* < 0.001). As a result, Hypotheses H2a, H2b, H2c, and H2f were confirmed. Conditional indirect analysis results for statistically significant causal relationships are presented in Table 6.

Therefore, following the bootstrapping, the conditional indirect effect was not verified for personality traits such as agreeableness (ME4) and openness to experiences (ME5). Hypotheses H2d and H2e were rejected. Additionally, the moderated mediating effect of perceived stress on reserve soldiers’ personality traits was confirmed by not including zero in the lower limit and the upper limit of the 95% confidence interval (CI) for extraversion (ME3, MMI = −0.022), openness to experiences (ME5, MMI = −0.013), and team cohesion (ME6, MMI = −0.053). Accordingly, Hypotheses H3c, H3e, and H3f were confirmed (see Table 6).

### 4.3. Evaluation of Moderated Mediation on Perceived Military Performance in Model 2 

First, the regression coefficients estimating the hardiness effect on personality traits and team cohesion were estimated by PROCESS macro v3.5 Model 14 (see Table A3, Appendix A). The result revealed that hardiness positively influenced conscientiousness (IV→ME1, β = 0.835, *p* < 0.001), emotional stability (IV→ME2, β = 0.682, *p* < 0.001), openness to experiences (IV→ME5, β = 0.268, *p* < 0.001), and team cohesion (IV→ME6, β = 0.460, *p* < 0.001) (see Table A3, Appendix A), and positively and directly influenced perceived military performance in this model (IV→DV, β = 0.121, *p* < 0.001, Table 5). Further, personality traits such as conscientiousness (ME1→DV, β = 0.176, *p* < 0.001, Table 5), openness to experiences (ME5→DV, β = 0.081, *p* < 0.001, Table 5), and team cohesion (ME6→DV, β = 0.235, *p* < 0.001, Table 5) positively and directly influenced perceived military performance in Model 2 (see Figure 1b). However, emotional stability (ME2→DV, β = −0.041, *p* < 0.05, Table 5) negatively and directly influenced perceived military performance. Moreover, the agreeableness personality trait (ME4→DV, β = −0.025, *p* = 0.260, Table 5) did not show significance in this model (see Table 5, Model 2). This seems to be that agreeableness and openness to experiences cannot mediate between hardiness and perceived military performance, but Hypotheses H1a, H1b, H1d, H1e, and H1f were confirmed. 

Furthermore, the results of the modeling allow us to confirm that the interaction of mediated variables and resilience significantly influenced perceived military performance—conscientiousness (ME1×MO2, F(1, 369) = 20.849, *p* < 0.001), emotional stability (ME2×MO2, F(1, 369) = 15.587, *p* < 0.001), openness to experiences (ME5×MO2, F(1, 369) = 9.365, *p* < 0.001), and team cohesion (ME6×MO2, F(1, 369) = 17.935, *p* < 0.001)—indicating that psychological resilience played a moderating role in the relationship between reserve soldiers’ personality traits, such as ME1, ME2, M5, and team cohesion (ME6). These results can confirm Hypotheses H4a, H4b, H4e, and H4f, that there is a moderating effect of resilience between mediated variables and perceived military performance (see Table 5, Model 2). The change in R^2^ with the addition of the interaction terms of personality traits, team cohesion, and resilience was statistically significant: ME1×MO2, ∆R^2^ = 0.019, *p* < 0.001; ME2×MO2, ∆R^2^ = 0.014, *p* < 0.001; ME5×MO2, ∆R^2^ = 0.008, *p* < 0.001; ME6×MO2, ∆R^2^ = 0.016, *p* < 0.001. This result means that when the interaction term is added, Model 2 will be able to account for more variance in perceived military performance (e.g., ME1 can extend for 1.9%, ME2 can extend for 1.4%, or ME6 can extend for 1.6%).

**Figure 2:**
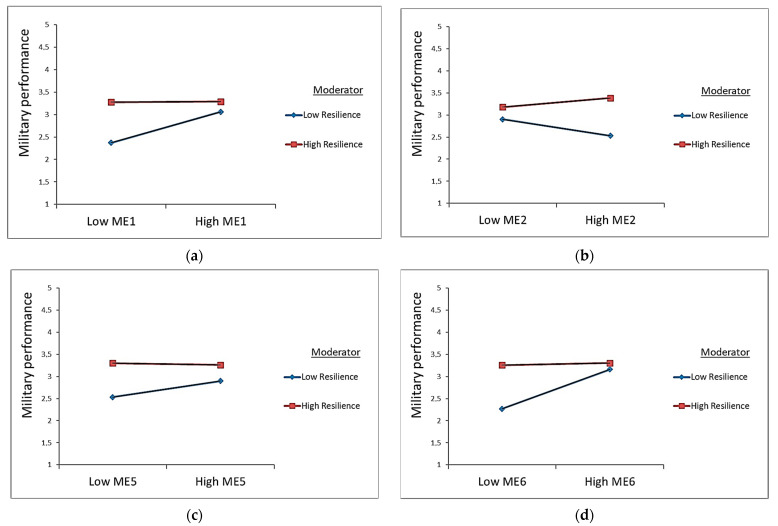
Statistically significant interaction effects on perceived military performance in Model 2: (**a**) interaction effect of conscientiousness (ME1) and resilience; (**b**) interaction effect of emotional stability (ME2) and resilience; (**c**) interaction effect of openness to experiences (ME5) and resilience; (**d**) interaction effect of team cohesion (ME6) and resilience.

The statistically significant moderated mediation indexes confirmed that psychological resilience has a moderating effect on the mediating role of reserve soldiers’ personality traits, such as conscientiousness (ME1, MMI = −0.141, 95% CI [−0.212, −0.082]), emotional stability (ME2, MMI = 0.100, 95% CI [0.046, 0.169]), openness to experiences (ME5, MMI = −0.029, 95% CI [−0.056, −0.006]), and team cohesion (ME6, MMI = −0.096, 95% CI [−0.156, −0.036]). Accordingly, Hypotheses H5a, H5b, H5e, and H5f were confirmed by not including zero in the lower limit and the upper limit of the 95% confidence interval. The statistically significant interaction effects of conscientiousness (ME1), emotional stability (ME2), openness to experiences (ME5), team cohesion (ME6), and resilience for perceived military performance are presented in Figure 2. 

In Model 2, reserve soldiers with less psychological resilience (M–1SD) were less likely to be confident in their perceived military performance when the levels of ME1, ME5, and ME6 were low rather than high (see Figure 2a,c,d). On the contrary, reservists with higher psychological resilience (M + 1SD) were more confident in their perceived military performance, regardless of whether the level of personality traits (ME1, ME2, and ME5) or team cohesion (ME6) was high or low. The specific situation appears for emotionally stable (ME2) reservists with low psychological resilience (M–1SD), and they were less confident about perceived military performance when ME2 levels were high rather than low (see Figure 2b).

Lastly, moderated mediating effects were also tested for whether study variables are moderated by marital status. For this modeling analysis, we used the PROCESS macro v3.5 Model 18, but the significance for marital status moderation was not confirmed.

### 4.4. Evaluation of Significance Area of the Moderating Variables in Model 1 and Model 2 

The significance area of the moderating variables (MO1 in Model 1 and MO2 in Model 2, see Figure 1) was examined and defined by Johnson–Neyman’s significance regions. The level of perceived stress (MO1) and psychological resilience (MO2) as moderators showed a statistically significant effect on reserve soldiers’ personality traits and team cohesion regions. The conducted analysis results that represent significant regions of perceived stress (MO1) and psychological resilience (MO2) are presented in Table 7.

The influence of hardiness on the perceived military performance results through personality traits varied in the interval [−1.43, 2.37] of perceived stress (MO1) scores. The extraversion trait (ME3) was significant in areas where the MO1 scores were less than 0.07 or greater 1.28; the openness to experiences trait (ME5) was significant only above the value of MO1; team cohesion (ME6) showed significance when the perceived stress scores (MO1) were less than −1.128. In other words, ME3, ME5, and ME6 mediate the relationship between hardiness and perceived military performance in areas where the perceived stress (MO1 in Model 1) scores were less than average for extraversion (ME3) and openness to experiences (ME5), and for team cohesion (ME6), the area of MO1 was as significant for the low stress values area as it was for the high stress values area. Our findings suggest that reserve soldiers who reported high levels of perceived stress relied more heavily on team cohesion, which, in turn, contributed to improvements in their perceived military performance. Further, it can be pointed out that average and high stress affect the expression of the openness to experiences trait (ME5). In contrast, even lower-than-average levels of perceived stress can affect the expression of the extraversion trait (ME3) (see Table 7, Model 1).

As for Model 2, where resilience (MO2) is the moderator (see Figure 1, Model 2), the personality traits and team cohesion mediate the relationship between hardiness and perceived military performance in areas where resilience scores are, for ME1, a resilience value less than 0.694; for ME2, a value less than 0.702; for ME5, a value less than 0.374; and for ME6, a value less than 0.694. This result means that reserve soldiers with high levels of hardiness have more ME1, ME2, and ME5 and are more focused on team cohesion (ME6), which improves their perceived military performance when their resilience is low (see Table 7, Model 2).

## 5. Discussion

Previous studies have shown that psychological hardiness affects the military performance of the tactical population in general [26] and military reservists in particular [18,63]. We extended these findings by adding the mediating effects of personality traits and team cohesion on this effect and moderating effects of perceived stress and resilience. Given that military training is characterized by a high intensity of stress [64], and at the same time, military training has a strong focus on building psychological resilience [22,65,66], we used perceived stress and psychological resilience as variables to better explain how hardiness affects performance in a stressful environment. Our study shows that studying multiple mediating–moderating effects provides a deeper understanding of how perceived stress and resilience affect the performance of soldiers than studying these effects alone. We discuss below the theoretical implications of our results, which can extend knowledge about human resource management in the military area, specifically for the development of a sustainable military reserve.

The first implication is related to hardiness and its effect on performance. As in previous studies [18,26,67], our results show a significant direct effect of hardiness on perceived military performance. Despite the fact that the conditional indirect effects of personality traits and team cohesion were significant, these mediators only partially mediated the significant variance between hardiness and performance for reserve soldiers. Therefore, our findings supported the hypothesis that personality traits and team cohesion mediate the relationships between hardiness and perceived military performance. Hardiness is an explanatory factor for why some soldiers pursue higher performance results and some do not. According to our findings, we can add that under high perceived stress, hardiness is a stronger predictor of performance than personality traits and team cohesion. This finding shows that hardiness has a significant impact not only on behavioral persistence [31] or retention in the military [26], but also on military performance. 

The second implication is related to perceived stress and its negative impact on perceived military performance. Consistent with previous studies on perceived stress in the military environment [68], our results show that perceived stress has a direct negative relationship with performance (r = –0.295, *p* < 0.001). On the basis of the results of the mediated moderation, we can provide a more detailed explanation. A negative effect of perceived stress weakens the positive effect of hardiness on performance when the person’s consciousness, emotional stability, and openness to experiences are weakly expressed and team cohesion is low. These results are similar to a meta-analysis [69,70], a large-scale population study [71], and a military-specific study [24], which found a negative association between adaptive personality traits and the level of perceived stress. 

The third implication is that high psychological resilience compensates for a lack of conscientiousness and emotional stability. Our result indicates that reservists with low psychological resilience were more at risk of showing lower performance due to their low levels of conscientiousness or openness to experiences. Conversely, if reservists have high resilience, then personality traits, whether expressed or not, will not diminish their perceived military performance. These results illustrate the classical definition of psychological resilience, which is described as a psychological ability or skill that could be applied in crises or stressful events [72,73]. These results lead not only to theoretical, but also practical implications. This suggests that more attention should be paid to specific training programs to improve the psychological resilience of reservists, as high psychological resilience is a good predictor of high performance in a military environment. 

The fourth implication is that very low levels of resilience could make soldiers act more within a team. This is especially true for team cohesion when hardiness is low. This finding is an extension of previous results on individual and team resilience, when these two variables were analyzed and developed separately [74]. 

The fifth implication is that psychological resilience has a stronger effect than perceived stress on performance. These findings support an added value of a range of resilience training programs that teach positive coping skills [15,29]. More specifically, our results indicate that the effect of personality traits (conscientiousness, emotional stability, and openness to experiences) and team cohesion on performance is stronger when resilience is higher. These results support the proposed Hypotheses H3a–3b and H3e–f. Stress during military exercises may not lead to high levels of perceived stress, as was found in some previous studies [28]. In this, the positive reframing happens [28], as psychological resilience is not only the ability to withstand stress, but also the ability to transform challenges into opportunities and the ability to recover [75]. In this context, even stressful training is effective (ensures high perceived military performance) if the resilience of reservists is developed.

Despite these implications, there are some limitations to consider. First, data collection was carried out at a single military base (place) and for one period of the year (January) when reservist military training was carried out. Different locations and seasons could change the results to some extent, as location and senescence have been indicated in more recent studies to have some, albeit minor, effect on the results [76]. This is particularly true for perceived stress studies, where adverse weather conditions create a more stressful environment [77]. Second, the research provides only evidence on the situation immediately after training. A longitudinal study that captures indicators at the beginning, middle, and end of the training would be more informative. Therefore, in the future, we plan to repeat this research by collecting data longitudinally. Third, our research sample comprised only young men, as only they were called for reserve training. This limits the interpretation of the research results to a broader category than just young men. Fourth, we must take into account the country factor as a limitation, as the research was carried out only in Lithuania (in the northeast of Europe). The level of perceived stress and other attitudinal indicators were found to vary according to geographical and cultural environments [57]. This also limits the interpretation of the study results in a wider geographical context. In particular, this research was conducted on self-reported measurements that could increase the overall variance error using a single-measurement system [71]. Fifth, only a single source of data was used to collect information on multiple variables; therefore, the common method variance (CMV) of bias could occur. Taking this into account, we estimated the interrater reliability of self-reported and instructor’s scores by the intraclass correlation coefficient (ICC). Although the high level of agreement between raters with an ICC coefficient of 0.804, which is considered almost ideal [78], can help overcome the common method variance (CMV) bias, it is important to acknowledge that this issue cannot be entirely disregarded. 

## 6. Conclusions

The study’s results revealed that psychological hardiness is a significant predictor of perceived military performance, and its impact is stronger under high perceived stress than that of personality traits and team cohesion. However, perceived stress has a direct negative effect on military performance, which weakens the positive effect of hardiness when personality traits and team cohesion are weakly expressed.

In addition, high psychological resilience compensates for a lack of conscientious-ness and emotional stability, which are personality traits that are typically associated with high performance. As a result, improving the psychological resilience of reservists is crucial for enhancing their military performance.

In contrast, very low levels of resilience could make soldiers act more within a team, particularly when hardiness is low. The findings of this study concluded that psychological resilience has a stronger effect on perceived military performance than perceived stress, suggesting the importance of promoting resilience training programs that can teach the soldiers positive coping skills.

The study results are valuable for understanding the determinants of the perceived military performance of reservists, i.e., people who are fully employed in the civilian sector and perform military training and missions as part of their civil duty. In addition, the results are valuable for the further development of psychological resilience training pro-grams designed to achieve greater sustainability of military reserve forces, as well as de-signed to help reserve soldiers achieve better work–life–duty balance.

## Figures and Tables

**Table 1:** Preliminary analysis results for the study variables.

Measurement Description	M (±SD)	CA	CR	AVE
Perceived military performance (14-item, 5-point Likert scale)				
DV: Performance	4.49 (0.63)	0.944	0.949	0.514
Military hardiness (11-item, 7-point Likert scale)				
IV: Hardiness		0.904	0.917	0.526
Personality traits (10-item, 7-point Likert scale)				
ME1: Conscientiousness	4.91 (1.15)	N/A		
ME2: Emotional stability	4.93 (1.19)	N/A		
ME3: Extraversion	4.16 (1.24)	N/A		
ME4: Agreeableness	4.57 (0.94)	N/A		
ME5: Openness to experiences	2.64 (1.38)	N/A		
Team cohesion (8-item, 7-point Likert scale)				
ME6: Team cohesion	4.32 (0.71)	0.774	0.899	0.527
Perceived stress during the training period (10-item, 5-point Likert scale)				
MO1: Stress	26.69 (5.87)	0.764	0.902	0.506
Psychological resilience (25-item, 5-point Likert scale)				
MO2: Resilience	4.14 (0.60)	0.756	0.911	0.508

Notes: N = 384 is the total number of the study dataset. M (±SD), mean +/− standard deviation. DV, dependent variable; IV, independent variable; mediators: ME1, ME2, ME3, ME4, ME5, ME6; Moderators: MO1, MO2. CR, Composite Reliability; AVE, Average Variance Extraction.

**Table 2:** Correlation between modeling variables.

Variable	Correlations									
	DV	IV	ME1	ME2	ME3	ME4	ME5	ME6	MO1	MO2
DV: Performance	--									
IV: Hardiness	0.554 **	--								
ME1: Conscientiousness	0.577 **	0.444 **	--							
ME2: Emotional stability	0.350 **	0.350 **	0.478 **	--						
ME3: Extraversion	−0.041	−0.095	−0.036	−0.152 **	--					
ME4: Agreeableness	−0.033	−0.110 *	0.030	0.065	0.033	--				
ME5: Openness to experiences	0.294 **	0.158 **	0.247 **	0.271 **	0.093	00.036	--			
ME6: Team cohesion	0.521 **	0.397 **	0.381 **	0.220 **	0.312 **	−0.003	0.220 **	--		
MO1: Perceived Stress	−0.295 **	−0.476 **	−0.276 **	−0.337 **	0.138 **	−0.043	−0.268 **	−0.171 **	--	
MO2: Resilience	0.511 **	0.292 **	0.308 **	0.198 **	−0.183 **	0.041	0.158 **	0.280 **	−0.342 **	--

Notes: N = 384 is the total number of the study dataset. Pearson’s correlation is significant at the * *p* < 0.05 and ** *p* < 0.01 levels (2-tailed).

**Table 3:** Mediator models with outcome variables of personality traits, such as conscientiousness, emotional stability, and extraversion under stress moderation.

	Step 1 Conscientiousness (ME1)			Step 2 Emotional Stability (ME2)			Step 3 Extraversion (ME3)		
		Coeff.	95% CI		Coeff.	95% CI		Coeff.	95% CI
Hardiness (IV)	$a_{11}$ →	0.726 *** (0.10)	[0.52, 0.93]	$a_{12}$ →	0.492 *** (0.11)	[0.28, 0.70]	$a_{13}$ →	−0.210 ^+^ (0.12)	[−0.44, 0.02]
Perceived Stress (MO1)	$a_{21}$ →	−0.103 (0.08)	[−0.27, 0.06]	$a_{22}$ →	−0.366 ***(0.09)	[−0.54, −0.19]	$a_{23}$ →	0.312 *** (0.10)	[0.12, 0.51]
Int_1 (IV × MO1)	$a_{31}$ →	0,098 (0.08)	[−0.06, 0.25]	$a_{32}$ →	−0.037 (0.08)	[−0.20, 0.13]	$a_{33}$ →	0.383 *** (0.09)	[0.20, 0.57]
Constant	${iM}_{1}$ →	0.021 (0.06)	[−0.09, 0.13]	${iM}_{2}$ →	−0.008 (0.06)	[−0.12, 0.11]	${iM}_{3}$ →	0.082 (0.06)	[−0.05, 0.21]
Model Summary	$R^{2}=0.206$ F(3, 380) = 32.901, *p* < 0.001			$R^{2}=0.161$ F(3, 380) = 24.246, *p* < 0.001			$R^{2}=0.063$ F(3, 380) = 8.502, *p* < 0.001		

Notes: N = 384 is the total number of the study dataset. Statistical significance: ^+^ *p* < 0.1, *** *p* < 0.001; 95% CI, bootstrap confidence interval of 95%.

**Table 4:** Mediator models with outcome variables of personality traits, such as agreeableness, openness to experiences, and team cohesion under stress moderation.

	Step 4 Agreeableness (ME4)			Step 5 Openness to Experiences (ME5)			Step 6 Team Cohesion (ME6)		
		Coeff.	95% CI		Coeff.	95% CI		Coeff.	95% CI
Hardiness (IV)	$a_{14}$→	−0.279 *** (0.09)	[−0.46, −0.10]	$a_{15}$→	0.138 (0.11)	[−0.07, 0.34]	$a_{16}$→	0.405 *** (0.06)	[0.28, 0.53]
Perceived Stress (MO1)	$a_{24}$→	−0.140 ^+^(0.08)	[−0.29, 0.01]	$a_{25}$→	−0.429 *** (0.09)	[−0.60, −0.26]	$a_{26}$→	0.078 ^+^(0.05)	[−0.03, 0.18]
Int_1 (IV×MO1)	$a_{34}$→	0.059 (0.07)	[−0.08, 0.20]	$a_{35}$→	−0.189 * (0.08)	[−0.35, −0.03]	$a_{36}$→	0.194 *** (0.05)	[0.10, 0.29]
Constant	${iM}_{4}$→	0.013 (0.05)	[−0.09, 0.11]	${iM}_{5}$→	−0.041 (0.06)	[−0.15, 0.07]	${iM}_{6}$→	0.042 (0.03)	[−0.03, 0.11]
Model Summary	$R^{2}=0.026$ F(3, 380) = 3.340, *p* < 0.05			$R^{2}=0.068$ F(3, 380) = 11.910, *p* < 0.001			$R^{2}=0.191$ F(3, 380) = 29.872, *p* < 0.001		

Notes: N = 384 is the total number of the study dataset. Statistical significance: ^+^ *p* < 0.1, * *p* < 0.05, *** *p* < 0.001; 95% CI, bootstrap confidence interval of 95%.

**Table 5:** Regression coefficients that estimate personality traits and team cohesion for the dependent variable of Model 1 and Model 2.

	Model 1 ^a^ Perceived Military Performance (DV)			Model 2 ^b^ Perceived Military Performance (DV)		
		Coeff.	95% CI		Coeff.	95% CI
Hardiness (IV)	$c^{\prime }$→	0.273 *** (0.04)	[0.19, 0.36]	$c^{\prime }$→	0.121 *** (0.04)	[0.04, 0.20]
Conscientiousness (ME1)	$b_{1}$→	0.172 *** (0.02)	[0.12, 0.22]	$b_{11}$→	0.176*** (0.02)	[0.13, 0.22]
Emotional stability (ME2)	$b_{2}$→	−0.041 * (0.02)	[−0.05, 0.04]	$b_{12}$→	−0.041 * (0.02)	[−0.08, −0.001]
Extraversion (ME3)	$b_{3}$→	−0.057 (0.02)	[−0.09, −0.02]	$b_{13}$→	−0.032 ^+^ (0.08)	[−0.07, 0.004]
Agreeableness (ME4)	$b_{4}$→	−0.008 (0.02)	[−0.06, 0.04]	$b_{14}$→	−0.025 (0.02)	[−0.06, 0.02]
Openness to experiences (ME5)	$b_{5}$→	0.069 *** (0.02)	[0.03, 0.11]	$b_{15}$→	0.081 *** (0.02)	[0.04, 0.12]
Team cohesion (ME6)	$b_{6}$→	0.273 *** (0.04)	[0.20, 0.35]	$b_{16}$→	0.235 *** (0.04)	[0.16, 0.31]
Resilience (MO2)				$b_{2}$→	0.284 *** (0.04)	[0.21, 0.36]
Int_1 (ME1 × MO2)				$b_{31}$→	−0.169 *** (0.04)	[−0.24, −0.10]
Int_2 (ME2 × MO2)				$b_{32}$→	0.146 *** (0.04)	[0.07, 0.22]
Int_3 (ME3 × MO2)				$b_{33}$→	−0.006 (0.03)	[−0.07, 0.06]
Int_4 (ME4 × MO2)				$b_{34}$→	0.009 (0.04)	[−0.06, 0.08]
Int_5 (ME5 × MO2)				$b_{35}$→	−0.100 *** (0.03)	[−0.16, −0.04]
Int_6 (ME6 × MO2)				$b_{36}$→	−0.210 *** (0.05)	[−0.31, −0.11]
Constant	iY→	2.456 *** (0.21)	[2.05, 2.87]	iY→	4.544 *** (0.02)	[4.50, 4.59]
Model Summary	$R^{2}=0.525$ F(7, 376) = 59.269, p < 0.001			$R^{2}=0.669$ F(14, 369) = 53.302, p < 0.001		

Notes: N = 384 is the total number of the study dataset. ^a^ Dependent variable model using Process v3.5 macro Model 7; ^b^ Dependent variable model using Process v3.5 macro Model 14. Statistical significance: ^+^ *p* < 0.1, * *p* < 0.05, *** *p* < 0.001; 95% CI, bootstrap confidence interval of 95%.

**Table 6:** Conditional indirect effects of perceived stress evaluated by using the PROCESS v3.5 macro-Model 7.

Moderator Perceived Stress (MO1)		Effect	BootSE	Boot LLCI	BootULCI
Conditional indirect effects (IV→ME1→DV)					
M-1SD	−0.738	0.112	0.028	0.062	0.170
M	0.000	0.125	0.022	0.082	0.170
M+1SD	0.738	0.137	0.022	0.094	0.180
Moderator perceived stress (MO1)		Effect	BootSE	Boot LLCI	BootULCI
Conditional indirect effects (IV→ME6→DV)					
M-1SD	−0.738	0.071	0.024	0.027	0.122
M	0.000	0.111	0.026	0.064	0.167
M+1SD	0.738	0.150	0.034	0.088	0.222
Conditional effects of the focal predictor (IV: Hardiness)					
Moderator perceived stress (MO1)		Effect (S.E.)	t	Boot LLCI	BootULCI
(IV→ME3)					
M-1SD	−0.738	−0.493(0.152)	−3.247 ***	−0.791	−0.194
M	0.000	−0.210(0.118)	−1.772 ^+^	−0.443	0.023
M+1SD	0.738	0.073(0.119)	0.064	−0.162	0.308
H3c: Moderated mediation index (MMI)		BootSE		Boot LLCI	BootULCI
−0.022		0.009		−0.043	−0.006
(IV→ME5)		Effect (S.E.)	t	Boot LLCI	BootULCI
M-1SD	−0.738	0.278(0.134)	2.065 *	0.013	0.542
M	0.000	0.138(0.105)	1.313	−0.069	0.344
M+1SD	0.738	−0.002(0.106)	−0.019	−0.210	0.206
H3e: Moderated mediation index (MMI)		BootSE		Boot LLCI	BootULCI
−0.013		0.007		−0.026	−0.001
(IV→ME6)		Effect (S.E.)	t	Boot LLCI	BootULCI
M-1SD	−0.738	0.262(0.081)	3.233 ***	0.103	0.421
M	0.000	0.405(0.063)	6.406 ***	0.281	0.529
M+1SD	0.738	0.548(0.064)	8.596 ***	0.423	0.673
H3f: Moderated mediation index (MMI)		BootSE		Boot LLCI	BootULCI
0.053		0.018		0.018	0.090

Note: Values for moderators self-efficacy (SEL) or socio-moral climate (SMC) are presented for ± one SD from the mean. Bootstrap sample size = 5000. Statistical significance: ^+^ *p* < 0.1, * *p* < 0.05, *** *p* < 0.001. Boot LLCI, boot lower level of confidence interval; Boot ULCI, upper level of confidence interval.

**Table 7:** Moderator values defined by Johnson–Neyman’s significance regions.

Model 1 Perceived Stress (MO1)	Interval [LLCI,ULCI]	Value	% below	% above
IV→MO1→ME3	[−1.43, 2.37]	−0.070	46.88	53.13
		1.278	94.79	5.21
IV→MO1→ME5	[−1.43, 2.37]	−0.588	26.04	73.96
IV→MO1→ME6	[−1.43, 2.37]	−1.128	5.21	94.79
Model 2 Psychological resilience (MO2)	Interval [LLCI,ULCI]	Value	% below	% above
ME1→ MO2 →DV	[−1.43, 0.86]	0.694	77.08	22.92
ME2→ MO2 →DV	[−1.43, 0.86]	0.010	58.33	41.67
		0.702	77.08	22.92
ME5→ MO2 →DV	[−1.43, 0.86]	0.374	72.92	27.08
ME6→ MO2 →DV	[−1.43, 0.86]	0.694	77.08	22.92

Note: Means were centered for the construction of products. Interval, value limit interval of 95% [LLCI, ULCI], where: LLCI, lower level of confidence interval; ULCI, upper level of confidence interval.

## Data Availability

Data supporting the reported results are archived in the National Open Access Research Data Archive (MIDAS) at www.midas.lt (accessed on 10 January 2023).

## Appendix A

**Table A1:** Hypothesis detailed.

Hypothesis ID	Hypothesis
H1	Reserve solders’ five personality traits, and their team cohesion will mediate the relationships between hardiness and perceived military performance:
H1a	Reserve soldiers’ levels of extraversion will mediate the relationship between hardiness and perceived military performance.
H1b	Reserve soldiers’ levels of agreeableness will mediate the relationship between hardiness and perceived military performance.
H1c	Reserve soldiers’ levels of conscientiousness will mediate the relationship between hardiness and perceived military performance.
H1d	Reserve soldiers’ levels of neuroticism will mediate the relationship between hardiness and perceived military performance.
H1e	Reserve soldiers’ levels of openness will mediate the relationship between hardiness and perceived military performance.
H1f	Reserve solders’ team cohesion will mediate the relationships between hardiness and perceived military performance.
H2	Perceived stress will play a moderating role in the relationship between hardiness and reserve soldiers’ personality traits and their team cohesion:
H2a	Perceived stress will play a moderating role in the relationship between reserve soldiers’ level of hardiness and the level of conscientiousness.
H2b	Perceived stress will play a moderating role in the relationship between reserve soldiers’ level of hardiness and the level of emotional stability.
H2c	Perceived stress will play a moderating role in the relationship between reserve soldiers’ level of hardiness and the level of extraversion.
H2d	Perceived stress will play a moderating role in the relationship between reserve soldiers’ level of hardiness and the level of agreeableness.
H2e	Perceived stress will play a moderating role in the relationship between reserve soldiers’ level of hardiness and the level of openness to experiences.
H2f	Perceived stress will play a moderating role in the relationship between reserve soldiers’ level of hardiness and the level of team cohesion.
H3	Perceived stress will have a moderating effect on the mediating role of reserve soldiers’ personality traits and team cohesion:
H3a	Perceived stress will have a moderating effect on the mediating role of reserve soldiers’ level of conscientiousness.
H3b	Perceived stress will have a moderating effect on the mediating role of reserve soldiers’ level of emotional stability.
H3c	Perceived stress will have a moderating effect on the mediating role of reserve soldiers’ level of extraversion.
H3d	Perceived stress will have a moderating effect on the mediating role of reserve soldiers’ level of agreeableness.
H3e	Perceived stress will have a moderating effect on the mediating role of reserve soldiers’ level of openness to experiences.
H3f	Perceived stress will have a moderating effect on the mediating role of reserve soldiers’ team cohesion.
H4	Perceived stress will have a moderating effect on the mediating role of reserve soldiers’ personality traits and team cohesion.
H4a	Perceived stress will have a moderating effect on the mediating role of reserve soldiers’ level of conscientiousness.
H4b	Perceived stress will have a moderating effect on the mediating role of reserve soldiers’ level of emotional stability.
H4c	Perceived stress will have a moderating effect on the mediating role of reserve soldiers’ level of extraversion.
H4d	Perceived stress will have a moderating effect on the mediating role of reserve soldiers’ level of agreeableness.
H4e	Perceived stress will have a moderating effect on the mediating role of reserve soldiers’ level of openness to experiences.
H4f	Perceived stress will have a moderating effect on the mediating role of reserve soldiers’ team cohesion.
H5	Psychological resilience has a moderating effect on the mediating role of reserve soldiers’ personality traits and team cohesion:
H5a	Psychological resilience has a moderating effect on the mediating role of reserve soldiers’ level of conscientiousness.
H5b	Psychological resilience has a moderating effect on the mediating role of reserve soldiers’ level of emotional stability.
H5c	Psychological resilience has a moderating effect on the mediating role of reserve soldiers’ level of extraversion.
H5d	Psychological resilience has a moderating effect on the mediating role of reserve soldiers’ level of agreeableness.
H5e	Psychological resilience has a moderating effect on the mediating role of reserve soldiers’ level of openness to experiences.
H5f	Psychological resilience has a moderating effect on the mediating role of reserve soldiers’ team cohesion.

**Table A2:** Demographic characteristics of the study respondents.

Demographic Characteristics	M(±SD) or N (%)
Gender (male%)	384 (100%)
Age (M;±SD)	29.53 (±4.91)
Marital status (N;%)	
1: Single	165 (43%)
2: Married	118(31%)
3: Live with a partner	61(16%)
4: Divorced	23(6%)
5: Widower	17(4%)
Education (N;%)	
1: Secondary	57 (14.8)
2: Professional	106 (27.6)
3: College	125 (32.6)
4: Baccalaureate	51 (13.3)
5: Master’s degree	45 (11.7)

Notes: N = 384 is the total number of the study dataset.

**Table A3:** Model 2, regression coefficients estimating hardiness effect on personality traits and team cohesion by PROCESS macro v3.5 Model 14.

	Step 1 Conscientiousness (ME1)			Step 2 Emotional Stability (ME2)			Step 3 Extraversion (ME3)		
		Coeff.	95% CI		Coeff.	95% CI		Coeff.	95% CI
Hardiness (IV)	$a_{11}$→	0.835 *** (0.09)	[0.67, 1.01]	$a_{12}$→	0.682 *** (0.09)	[0.50, 0.87]	$a_{13}$→	−0.191 ^+^ (0.10)	[−0.39, 0.01]
Constant	${iM}_{1}$→	−3.563 *** (0.37)	[−4.29, −2.83]	${iM}_{2}$→	−2.911 (0.40)	[−3.70, −2.12]	${iM}_{3}$→	0.816 (0.44)	[−0.06, 1.69]
Model Summary	$R^{2}=0.198$ F(1, 382) = 94.026, *p* < 0.001			$R^{2}=0.123$ F(1, 382) = 53.494, *p* < 0.001			$R^{2}=0.009$ F(1, 382) = 3.461, *p* = 0.064		
	Step 4 Agreeableness (ME4)			Step 5 Openness to Experiences (ME5)			Step 6 Team Cohesion (ME6)		
		Coeff.	95% CI		Coeff.	95% CI		Coeff.	95% CI
Hardiness (IV)	$a_{14}$→	−0.169 * (0.08)	[−0.32, −0.02]	$a_{15}$→	0.268 *** (0.09)	[0.11, 0.47]	$a_{16}$→	0.460 *** (0.05)	[0.35, 0.57]
Constant	${iM}_{4}$→	0.720 * (0.34)	[0.06, 1.38]	${iM}_{5}$→	−1.221 ** (0.39)	[−2.00, −0.45]	${iM}_{6}$→	−1.961 *** (0.24)	[−2.42, −1.50]
Model Summary	$R^{2}=0.012$ F(1, 382) = 4.691, *p* < 0.05			$R^{2}=0.025$ F(1, 382) = 9.779, *p* < 0.01			$R^{2}=0.157$ F(1, 382) = 29.872, *p* < 0.001		

Notes: N = 384 is the total number of the study dataset. Statistical significance: ^+^ *p* < 0.1, * *p* < 0.05, ** *p* < 0.01, *** *p* < 0.001; 95% CI, bootstrap confidence interval of 95%.
